# Why all lawyers must study entomology

**DOI:** 10.1093/aesa/saae019

**Published:** 2024-08-14

**Authors:** David W Onstad

**Affiliations:** Editor-in-Chief

In late June of 2024, the United States Supreme Court ruled that the courts (meaning judges) must be the interpreters when laws passed by Congress are converted to regulations. For the past 40 years, legal precedent has held that federal courts should generally defer to reasonable interpretations by agency experts when laws are ambiguous (known as “Chevron deference” from the 1984 deciding case). The new ruling gives judges the authority to potentially override or disregard the input of agency subject–matter experts and exert their own decisions without room for objection.

For a concise description of these issues about law and judging that have developed over past decades, read Judge David Tatel’s (2024) chapter titled “The Future of the Planet.” He discusses several important cases concerning environmental regulation. He addresses uncertainty, ambiguity, reasonableness, and the value of deference to scientific regulation. The trend he perceived was a predictor of what happened in the ruling by the Supreme Court a few weeks after the publication of his book. The new ruling opens the door for judges to exert personal influence on rulings now that the precedent of deference to agency experts has been overturned.

In the past, deference to agency experts was the norm and likely ensured that the people closest to the science were the ones to weigh in on the evidence for a particular decision. Barnett and Walker (2017) found that the courts applied deference to regulatory-agency experts 77% of the time. For cases concerning agricultural regulation, the agencies won 67% of all cases and 77% of cases in which the deference to agency experts was applied.

Science is dynamic. Our knowledge and hypotheses improve all the time. Thus, regulations should improve as the best available science changes. It is hard to believe that Congress wants to, or even can, reconsider every law that protects beneficial arthropods and produces safer pest management as changes in science, the environment, human population density, and technology occur.

So, why is any of this of concern to entomologists? We know that a lot of our work is affected by policies … from endangered arthropod species, to pest management application, to monitoring for invasive species. Here are two examples of the type of scientific guidance from the Centers for Disease Control and Prevention, the Environmental Protection Agency or the Fish and Wildlife Service that will be interpreted by judges. (i) A citizen group could challenge the authority of a municipality to do an aerial spray for mosquito control to prevent West Nile virus, or Eastern equine encephalitis. (ii) A citizen could challenge thresholds for classifying threatened and endangered arthropods such as total number of remaining individuals, number of distinct populations, the results of a population viability model analysis. We all know that good science can (and should) be challenged, but we typically think that these debates will be carried out over time in scientific settings, not courts.

The Supreme Court now believes that the experts should be called into courts to testify about their ideas. In court, one side calls its experts as witnesses and the other side calls its set. But if 90% of entomologists believe one hypothesis and 10% believe something else, science typically emphasizes the conclusion drawn by the 90%. However, in court, the judge/jury hears from equal numbers of scientists from each side of the debate. As these decisions are made in lower courts nationwide, it could create a patchwork of varying decisions resulting in chaos in knowing what is legal in different states, or even counties in the nation.

Instead of spending money on litigation shouldn’t we use the money for more research to improve science? An estimated cost of litigating a case all the way through the system to the Supreme Court is over $1 million (Washington Post 2011). This is likely an underestimate for future cases. There are also significant opportunity costs to this approach to entomological regulations. If entomologists are expected to spend much more time preparing testimony and then testifying in court, much less time is available to learn more and explain the science that will improve regulations. One activity that will suffer the most is manuscript reviewing. If entomologists don’t provide rigorous reviews of manuscripts, judges and lawyers will have more to criticize about our science and our contributions to regulations.

As we think about how these cases could play out, it’s clear there may be a need for entomologists to speak up and attempt to educate lawyers and judges. This situation highlights the value of the Entomological Society of America’s Science Policy Committee in (i) communicating with Congress and the Executive Branch and (ii) educating ESA members about policy-making and their application.

If we want justice to prevail as more and more science-based cases appear before the courts, entomologists need to engage with those who plan to become lawyers. (It may be impossible to educate them once they become judges.) Of course, an alternative would be for entomologists to take a course on regulatory law. At a minimum, entomologists need to recognize the importance of science policy.

I thank Politico (2024) for enlightening me about the Supreme Court decision and leading me to the article by Barnett and Walker.
